# Opportunities for Digital Health to Support Early Psychosis Care in Ghana: Qualitative Study Among Patients, Caregivers, and Clinicians

**DOI:** 10.2196/85046

**Published:** 2026-04-20

**Authors:** Anna Larsen, Joel Agorinya, Alexa Beaulieu, Dzifa Abra Attah, Justin Tauscher, Seth Asafo, Benjamin Buck, Emmanuel Quame Sottie, Ella DeVries, Audrey Florence Okyere, Millicent Ampaw, Caleb Agyemang Duah, Angela Ofori-Atta, Kwadwo Obeng, Dror Ben-Zeev

**Affiliations:** 1 Department of Psychiatry and Behavioral Sciences, School of Medicine, University of Washington, 1959 NE Pacific Street, Seattle, WA, 98195, United States, 1 2063342783; 2 Accra Psychiatric Hospital, Accra, Ghana; 3 Department of Psychiatry, School of Medicine, University of Ghana, Accra, Ghana; 4 Department of Psychiatry, University of North Carolina at Chapel Hill, Chapel Hill, NC, United States

**Keywords:** early psychosis, digital health, technology serious mental illness, Ghana, West Africa, qualitative, patients, caregivers, clinicians

## Abstract

**Background:**

Youth experiencing early psychosis in West Africa often face delays in accessing evidence-based treatment. Digital mental health interventions may offer an acceptable and scalable approach to improve access to early psychosis care in West Africa; however, few data exist on the experiences and perspectives of patients with early psychosis and their caregivers to inform digital intervention development.

**Objective:**

This study aims to explore current experiences of early psychosis care, identify barriers and facilitators to in-person early psychosis care within health facilities, and identify opportunities for digital interventions to support patients with early psychosis and caregivers in Ghana.

**Methods:**

We conducted qualitative focus group discussions among patients with early psychosis, their caregivers, and their mental health care providers recruited at Accra Psychiatric Hospital in Accra, Ghana. Trained qualitative researchers facilitated discussions using a structured qualitative interview guide, exploring current care practices for early psychosis in Ghana, barriers and facilitators to facility-based care, and perceptions of digital mental health interventions. Transcripts were translated, transcribed, and analyzed thematically using a hybrid inductive and deductive approach grounded in the theoretical framework of acceptability.

**Results:**

Overall, we conducted 4 focus group discussions (N=31) among 7 patients with early psychosis (median age 28, IQR 21‐41 years), 6 caregivers (median age 58, IQR 29‐34 years), and 18 clinicians (median age 30, IQR 29‐34 years). Participants described current early psychosis care practices in Ghana, including seeking spiritual and traditional healing, the dearth of information and resources about psychosis, and the integral role of caregivers in facilitating treatment engagement and continuation (often at the cost of caregiver mental distress and burnout). Common barriers to facility-based mental health care included stigma associated with mental illness, lack of prior knowledge about early psychosis and treatment options, and practical constraints (eg, financial, logistical, and health care system limitations). Motivating factors for facility-based care included success stories from community members and strong rapport and trust in mental health clinicians. Technology (eg, mobile phones, laptops, radio, and television) was commonly used among participants in typical daily tasks, health information seeking, and stress reduction. Participants expressed support for digital tools that could deliver psychoeducation about early psychosis, support treatment adherence, and extend patient-provider communication between clinic visits.

**Conclusions:**

Digital mental health interventions have the potential to complement facility-based early psychosis services in Ghana by addressing misinformation, reducing access barriers, and supporting caregiver roles. These qualitative findings inform potential integration points, content, attributes, and strengths of digital modalities that could be leveraged to support patients with early psychosis and their caregivers in Ghana.

## Introduction

Mental health service coverage remains low across West Africa [1,2], where individuals with untreated serious mental illness face increased risk of poverty, exclusion, neglect, and abuse [3,4]. Early psychosis is defined as the period when a person first begins to experience hallucinations, delusions, or difficulty distinguishing reality [5]. This period represents a key window for intervention. Reducing the duration of untreated psychosis can alter the course of illness toward better psychiatric and social outcomes [5,6]. Evidence-based interventions such as antipsychotic medications and cognitive behavioral therapy for psychosis are most effective when introduced early, reducing symptom severity, risk of hospitalization, and functional impairment [7-9]. Strong community ties and family support systems are assets in low- and middle-income country (LMIC) settings; there is evidence that recovery outcomes following early psychosis treatment may be especially strong in LMICs [10]. Yet in West Africa, few youths with early psychosis receive these interventions since availability is scarce [11]. Systematic estimates of early psychosis care uptake are not available for this region; however, a cross-sectional survey in Ethiopia identified that 40% of people with psychosis received treatment for a current episode, and that more than 70% of them did not receive “minimally adequate care” (at least 4 follow-up care visits with medication monitoring) [12].

In many cases, people with early psychosis and their caregivers first seek care from religious or traditional healers, delaying access to psychiatric or psychological treatment [13,14]. Beliefs about the spiritual origin of symptoms—such as witchcraft or prophetic calling (the belief that psychotic symptoms are spiritual messages from a deity) [15]—alongside high levels of stigma and health care system barriers limit access to care in formal health care settings. Long distances to clinics, understaffed facilities, and extended wait times further discourage service use, extend the duration of untreated psychosis, and have made it difficult to integrate mental health care into primary care systems [16]. Even when individuals are diagnosed in a formal psychiatric setting, medication discontinuation and disengagement from care are common among patients with early psychosis globally [17]. The World Health Organization recommends family-inclusive models in LMICs [18] that align with local norms of reciprocal caregiving [19] and leverage the central role played by caregivers in the recovery and social integration of their loved ones with early psychosis [20]. In West Africa, however, families often have little access to information or support to fulfill this role [19].

Addressing unmet needs among individuals and families affected by early psychosis in West Africa requires novel approaches that overcome known care-seeking barriers [21,22]. Mobile and digital tools (eg, mobile- and internet-enabled tools such as SMS text messaging and smartphone apps) may offer one way to increase access to mental health support [21], particularly given the high levels of mobile phone ownership (>75%) reported across many countries in the region [23]. Ghana is increasingly recognized as a West African leader in digital innovation, particularly for improving health infrastructure [24]. Ghana also has an advanced telecommunications infrastructure and a longstanding, albeit underresourced, mental health care system. Digital tools for mental health can leverage widely available infrastructure—such as mobile phones and internet access—and may reduce barriers related to transportation, wait times, and concerns about privacy or stigma that impede access to evidence-based early psychosis psychological treatment.

Mobile health tools for psychological support have been shown in high-income settings to improve symptoms and reduce relapse for youth with early psychosis and to help families communicate about mental health [25-28]. Digital tools for behavioral health are expanding rapidly in African contexts, particularly in areas such as HIV care and maternal-child health [29]. Such tools offer scalable support for the treatment of early psychosis in low-resource settings by incorporating educational and caregiver-involvement features. Yet, there are currently no digital resources developed or adapted for people experiencing early psychosis and their caregivers in the African context, despite growing support from the World Health Organization for digital mental health interventions in LMICs [30].

To explore current care practices for early psychosis in Ghana, barriers and facilitators to mental health service use, and opportunities for digital approaches to address early psychosis, we conducted a qualitative study among people experiencing early psychosis, their caregivers, and mental health care providers.

## Methods

### Study Design, Participants, and Setting

To qualitatively understand experiences of those impacted by early psychosis, current care practices for early psychosis in Ghana, barriers and facilitators to accessing in-person early psychosis care in hospitals and clinics, and opportunities for digital mental health to address early psychosis, we conducted focus groups with patients with early psychosis, their caregivers, and mental health care providers at Accra Psychiatric Hospital (APH). This facility is Ghana’s national psychiatric referral hospital. It provides services to 28,000 outpatients per year and serves up to 300 inpatients at a time, making it the largest psychiatric hospital in Ghana. Patients and their families often travel long distances within Ghana and the surrounding region to receive psychiatric services at APH. Ghana’s mental health care system is situated within a multitiered public sector health care system comprised of community, district, regional, and national-level health facilities. Mental health care professionals are situated within each level, predominantly master’s-level trained practitioners. Typical pharmacological treatments include injectable and oral first-generation antipsychotics. Psychotherapeutic modalities such as supportive counseling are provided, which sometimes includes manualized cognitive behavioral therapy. Ghana’s National Health Insurance Scheme includes coverage for mental health services; however, implementation does not always consistently cover these services, leading to out-of-pocket expenses.

### Study Procedures

We identified patient and caregiver participants for potential participation in focus group discussions through purposive sampling, contacting families participating in a pilot program at APH for early psychosis. This pilot program aimed to provide coordinated multispecialty and assertive community outreach services to individuals experiencing early psychosis. Early psychosis was characterized as the onset of psychotic symptoms within the previous 5 years, with no continuous pharmacological treatment of more than 3 consecutive months. To assess interest in study participation among patients with early psychosis and caregivers enrolled in the early psychosis pilot program, trained research staff (psychiatric residents training and practicing at APH) reached out to the caregivers of patients identified with early psychosis via telephone. All patients and caregivers enrolled in the pilot program at APH were contacted. Participation by patients and caregivers was independent, with no requirement that both be involved. We held 1 focus group for patients with early psychosis and 1 for caregivers. Focus groups were conducted at APH and lasted about 1 hour.

Mental health care providers were eligible for participation in focus group discussions if they were actively providing mental health care to individuals experiencing early psychosis at APH. We recruited providers by inviting all APH mental health clinicians providing early psychosis care (eg, clinical psychologists, psychiatrists, and psychiatric residents) to a seminar on opportunities for digital mental health to support early psychosis care. Providers interested in contributing provided contact information and were scheduled into 1 of 2 focus group discussions based on clinical schedule and workload. To accommodate interest and optimize inclusion of provider experiences, we held two 1-hour focus groups at APH for early psychosis clinicians. Focus group sessions were audio-recorded, translated (from a mix of English and Twi into English), and transcribed.

Data on demographic characteristics were collected for each participant using REDCap (Research Electronic Data Capture; Vanderbilt University) electronic questionnaires on tablets by trained data assessors. Qualitative researchers (DAA and AL) used a structured interview guide that was informed by the theoretical framework of acceptability [31] to inquire about the topics of interest (experiences of those impacted by early psychosis, current care practices for early psychosis in Ghana, barriers and facilitators to facility-based early psychosis care, and opportunities for digital mental health interventions). Focus group discussions were conducted in a private room away from clinical activity on the APH campus. Discussions were carried out in English with translation into Twi by translators (AFO and CAD) fluent in both languages. Accra-based researchers cofacilitated the discussions and reviewed the data to mitigate power imbalances and ensure culturally sensitive interpretations.

### Qualitative Analysis

We conducted a thematic analysis of the focus group transcripts using a mixed inductive and deductive approach. Thematic analysis involves identifying, organizing, and interpreting patterns of meaning within qualitative data [32]. This flexible method follows a structured process, including data familiarization, coding, theme development, review, and refinement. We followed a mixed deductive (ie, predefined codes) and inductive (ie, codes emerging from data) approach to facilitate identification of themes emerging from participants’ narratives while also attending to topics of interest informed by our interview guide and relevant literature [33].

#### Coding Process

Transcripts were first reviewed for accuracy and completeness following translation from Twi to English (AFO, MA, and DAA). A structured codebook was developed collaboratively by the 3-person coding team (AL, AB, and ED), all researchers from the University of Washington.

The coding team consisted of a Research Manager with a Master of Public Health degree and a background in global mental health (AB), a Research Coordinator with a Bachelor’s degree in Psychology (ED), and a research faculty member with a doctoral degree in epidemiology and a background in global mental health (AL). Two team members (AB and AL) had 3 years of experience conducting research in Ghana’s mental health care system; the other member (ED) did not have prior experience working in Ghana. Researchers from APH (JA and KO) and the University of Ghana (DAA and SA) reviewed and enhanced cultural understanding and contextual relevance of the codebook and later interpretation of the data, improving trustworthiness of the analysis [34]. The codebook drew on both the interview guide (deductive codes) and initial readings of the transcripts (inductive codes), with detailed definitions for each code and subcode to ensure consistent application across coders. To ensure the rigor of our analysis, we used multiple strategies, including maintaining an audit trail of coding decisions and codebook revisions, systematic double-coding of all transcripts, and regular team meetings to discuss emerging findings and resolve coding discrepancies. The structured codebook facilitated consistency in coding application across team members [34].

Coding was conducted systematically using Dedoose qualitative analysis software (version 10.0.34; SocioCultural Research Consultants, LLC). Two coders (AB and ED) independently coded 30% of the transcripts to pilot the code application. Coders met to discuss discrepancies in initial coding, clarify definitions, and agree on application protocols. Following this pilot coding, each transcript was independently coded using the structured codebook. The coding team met regularly to discuss code definitions, resolve discrepancies, and refine the codebook based on emerging patterns in the data. Disagreements between coders were resolved through discussion, with a third team member (AL) serving as an adjudicator when consensus could not be reached. This approach facilitated a robust and consistent method to ensure coding reliability and consistency across the dataset [35].

#### Theme Development

Following systematic coding, the team reviewed coded segments and grouped related codes into preliminary themes. These themes were iteratively reviewed, refined, and, where necessary, collapsed or expanded to ensure they were distinct, coherent, and well-supported by the data. Coding memos were used throughout the process to document the rationale for theme development and any changes made during the analytic process. Final themes were agreed upon by the full coding team and illustrated with representative quotations from the transcripts.

### Ethical Considerations

Verbal consent was obtained from all individuals before participation, as this study was deemed sufficiently low risk to allow verbal consent. Study participants were compensated with 140 Ghanaian Cedis (~US $10)—an amount comparable to covering their transportation costs and time to and from the study setting. This amount did not represent undue influence in this economic and cultural context. Ethical approval for the study was granted by the University of Washington Institutional Review Board (STUDY00020314) and the Ethics Review Committee of APH (APH-ERC 00020/24). Participants were provided with a copy of the informed consent form, as desired, which made clear their right to withdraw from the study at any time. Privacy and confidentiality were protected for all study participants. Before study enrollment, team members clarified that participation was voluntary and unrelated to clinical services.

## Results

### Description of Populations

Overall, 31 participants engaged in focus group discussions to inform the development of a digital support tool for individuals impacted by early psychosis in Ghana. Seven patients with early psychosis, 6 of their caregivers, and 18 clinicians participated. Among patients with early psychosis, the median age was 28 (IQR 21‐41) years (Table 1). Clinicians were slightly older, with a median age of 30 (IQR 29‐34) years. Caregivers had a median age of 58 (IQR 54‐68) years. A third of patients with early psychosis and their caregivers were female (patients: n=2, 29% and caregivers: n=2, 33%), while our sample of clinicians had an equal distribution of men and women.

**Table 1.** Characteristics of focus group discussion participants impacted by early psychosis in Ghana.

Characteristic	Patients with early psychosis (n=7)	Early psychosis caregivers (n=6)	Clinicians (n=18)
Age (years), median (IQR)	28 (21-41)	58 (54-68)	30 (29-34)
Sex, n (%)			
Female	2 (29)	2 (33)	9 (50)
Male	5 (71)	4 (67)	9 (50)
Marital status, n (%)			
Married/cohabiting	1 (14)	5 (83)	8 (44)
Divorced	0 (0)	1 (17)	1 (6)
Never married	6 (86)	0 (0)	9 (50)
Highest educational attainment, n (%)			
Primary	1 (14)	1 (17)	0 (0)
Secondary	3 (43)	4 (67)	0 (0)
Tertiary	3 (43)	0 (0)	0 (0)
University	0 (0)	0 (0)	10 (56)
Graduate	0 (0)	0 (0)	8 (44)
Other (eg, below primary)	0 (0)	1 (17)	0 (0)
Literacy, n (%)			
English only	5 (71)	4 (67)	6 (33)
Twi only	0 (0)	1 (17)	0 (0)
Both	2 (29)	0 (0)	12 (67)
Neither	0 (0)	1 (17)	0 (0)
Religion, n (%)			
Christian	7 (100)	6 (100)	12 (67)
Islamic	0 (0)	0 (0)	6 (33)
Residential setting, n (%)			
Urban (city)	7 (100)	6 (100)	17 (94)
Peri-urban (town)	0 (0)	0 (0)	1 (6)
Regularly employed	1 (14)	5 (83)	18 (100)
Job position, n (%)			
Psychiatrist	—^a^	—	1 (6)
Clinical psychologist	—	—	1 (6)
Psychiatric resident	—	—	9 (50)
Medical officer/clinical psychology trainee	—	—	7 (39)
Travel time to Accra Psychiatric Hospital (minutes), median (IQR)	60 (60-120)	60 (45-120)	—

a Not applicable.

The most common role among clinician participants was Psychiatric Resident (n=9, 50%)—many of the clinicians providing direct care to patients with psychosis are Psychiatric Residents completing their training at APH. The second most common role was Medical Officer or Clinical Psychology trainee (n=7, 39%), indicating that our sample of clinicians represented relatively early-career professionals, which is well-aligned with the demographics of practicing clinicians within APH and the Ghana National Mental Health System.

Only 1 early psychosis patient was married (n=1, 14%), while caregivers were predominantly married (n=5, 83%), and nearly half of clinicians were married (n=8, 44%). The majority of patients with early psychosis had completed secondary or tertiary education (n=6, 86%), a lower proportion of caregivers had achieved more than primary education (n=4, 67%), and all clinicians had achieved university education or higher (n=18, 100%). Only 1 early psychosis patient was regularly employed (n=1, 14%), while most caregivers (n=5, 83%) were employed. Patients with early psychosis and their caregivers lived on average 1 hour away from APH. Demographic characteristics of these study participants are similar to those in a survey of 256 patients with early psychosis and their caregivers conducted by our team at APH, demonstrating that this sample is representative of this population visiting APH [36].

### Current Care Practices for Early Psychosis in Ghana

During focus group discussions, participants described the current status of care practices for early psychosis in Ghana, including actions taken to address early psychosis, sources of early psychosis support and information, and how caregivers are involved in early psychosis care. Additional representative quotations regarding current care practices are provided in Table 2.

**Table 2.** Illustrative quotations on current care practices for early psychosis in Ghana.

Category	Representative quotations
Actions taken to address early psychosis	
Consulting others	“The next day, she got really aggressive and took her bag and said she was leaving. So I had people help me stop her. Her brother who was playing football in the neighborhood came home and suggested we take her to the Polyclinic but my sister insisted we bring her here because my daughter was too aggressive. By God’s grace, we we’re able to get her here safely.” (Caregiver) “I was there one day when a friend of mine told me he knew someone who struggled with similar issues and when the person was brought to this hospital, that person got better and is even married now. I then decided to discuss it with my child’s mother so we get a solution. She also agreed and we brought the child to this hospital. We were so lucky it was Dr. [Name] we met when we came. Dr. [Name] saw the symptoms my son was experiencing.” (Caregiver) “For me, it was my pastor who helped me because the church was the first place I ran to when my child’s condition worsened. He prayed for us but after three days his symptoms got worse again. I called him to inform him that was when he told me he’d seen my son around the church and then he cautioned me to get him to this hospital. My pastor is also a doctor. So I brought my son here. I see the improvements now. And it’s all because of his help and that’s of the staff in this facility.” (Caregiver)
Religious, spiritual, and traditional approaches	“So, if they manage to take the clients there, and then say, those that have suffered acute psychosis, and maybe, like, after a day or so, they see that they’re improving, they’d rather take them into pastors or churches. They would take them to religious leaders or traditional things. They’d rather take them to those places.” (Clinician) “…when the child’s condition first came about, his mother took him to some churches and prayer camps which didn’t help. I didn’t like those places because Some of the churches can deceive you and ask you to bring some money, buy all sorts of things which will result in no improvement in their health.” (Caregiver) “Mostly they are brought in by relatives and that’s usually after they’ve sought help from other spiritual camps and you know some are attributed to witchcraft and all that. So I think most of the time they come in with family or they are referred err maybe they present to hospitals on account of different things then they refer to us. But it’s very unusual for you to see a patient with psychosis come in voluntarily.” (Clinician)
Facility-based care	“At first uh my parents, they didn’t believe what I was going through and uh I used force. I used to tell them anything with force before they told me to go to the hospital. So, I went to a clinic at my place called Zion. Zion Hospital. So they the doctor referred me to see a psychiatrist. So that’s when I realized that I have to come to the parents and let them know where to go. So that’s when we came to this place.” (Patient) “I also think that some of them also seek regular health care. They actually do often. I remember when I was doing my obstetric rotation, we had a patient come in complaining about, that was postpartum, so she had some form of postpartum psychosis. She actually walked in with her husband and talked about it. So we had, like, this whole session. And we had a couple of the women also opening up after that. So I think some of them also seek regular health care.” (Clinician) “She started hearing voices and cries of animals for four continuous days. She felt it was abnormal. So she informed her sister that ‘what I'm hearing is not normal. I want to go to a general hospital.’ So on their way, they brought her here. So she felt like they had betrayed her.” (Clinician)
Sources of early psychosis support and information	
Unhelpful advice or information	“In my case, when the condition started, I went to my pastor to pray for me and he said she wasn’t sick. My pastor said there was nothing wrong with her rather, she just enjoyed going out with her friends. And I told him I know my daughter. I can tell she’s sick. So he prayed for her and said there was no need to take her to the hospital but when I saw where things were going, I brought her to the hospital.” (Caregiver) “You see uh, how do I put this, You get a lot of input from different people when something happens. What you hear is what causes you to make it move. You (claps) don’t know what’s happening. So the incident would have to first happen for you to know the steps you must take. For instance, you can get two or three great advises from about 10 people. That’s how we roamed through different prayer camps before arriving at our last resort which is this hospital.” (Caregiver)
Helpful advice or information	“When the doctor diagnosed me that I had schizophrenia, I went to research about the sickness, schizophrenia. They said it’s a mental illness caused during the adolescence age of a, of an individual. So, I had knowledge about the sickness. Even if it wasn’t for the doctor, like I hadn’t heard of that sickness before. And it has helped so, anytime I can see someone suffering from that, I can know. I’m not a doctor but I can also give the maybe the person is also suffering from schizophrenia.” (Patient) “So when you also inform them about some of the success stories with other clients who are engaged, like they are doing okay in their work, they are they get hopeful or they know that oh they can also do something about their lives and their life doesn’t mean that once they get out of this illness, then they are not going to amount to erm anything. So I think that’s when when they get resolution in their symptoms is also the success story.” (Clinician)
Role and toll of caregivers in early psychosis care	
Encouraging formal health system care and treatment adherence	“In the year 2019‐2020, I was with a friend and I involved myself into drug abuse, was into this codine, raffinol, so um during that period, I start to have dreams. Dreams and nightmares. My dreams was very rough. So I didn’t understand myself. So it’s something that I started to tell people about my story. Nobody could help me. So I came here with my dad. That’s when they prescribed medicine for me.” (Patient) “As a parent, you would have to do everything possible to get your child the right treatment, even if you have to borrow money for it. You’ll now have to make sure your child sees how much you love them so that their situation wouldn’t get worse. Don’t listen to what people will say about the whole thing, rather heed to the directions of the doctors and you’ll get the results you desire.” (Caregiver) “The few issues we’ve had are the ones with very strong delusions, the systematized delusions, and those that poor insight is actually part of the diagnosis. You see that in some schizophrenics, that generally they have a very poor insight. So getting them to understand the condition and adherence on medication is a challenge. Like the…if the family members get to understand this and would help them to be taking their medications, providing them with the support to come for reviews, I mean eventually, we are yet to get there but eventually we will see a change in their conditions too.” (Clinician)
Comforting, listening, talking, and strategizing	“So when your child starts experiencing symptoms, truth be told, your neighbors will have different opinions about what is happening. Rumors ranging from drug abuse to thefts, your neighbors will say lots of different things. You, the parent, would now be the one to better explain the situation to the neighbors, well enough to keep your child safe and happy.” (Caregiver) “What helped us decide to take action was love. And a parent wouldn’t just stand by while their child suffers so you’ll have to act fast to get them the right care. Health care is what we’re trying to get for our loved ones.” (Caregiver)
Material support (housing, transportation, and financial)	“Anytime the doctors call my mother that maybe we’ll be having a review, maybe this date or a specific date, she comes to drop me before she goes to work. So as for that one, anytime we have an appointment here, she- makes she takes her time to drop me before she goes to work. So that’s the support I receive for now.” (Patient) “This is the phone I use. Only this one. I can’t go buy an expensive phone when my child has a mental health disorder. I spend Gh¢ 70 everyday, how can I spare money you buy a phone worth over Gh¢ 1500. I use this same phone to update two of my friends about my son’s condition and ask them to help him find a job.” (Caregiver) “As for me… (03) when my child got sick, I used up most of my money. I sold my fridge, two TV sets, and the smaller TV set we were using broke about a week ago…It’s just there. So the TV set we watch to get our mind off things is now broken. My son is now distraught about the TV. He keeps asking me to help him fix it and I can’t. So all these are some of the problems.” (Caregiver)
Mental stress, burnout, and burden on caregivers	“So if you don’t have God to look up to (kisses teeth) you’d start hating the child. Because its embarrassing seeing your child walking about and picking up trash by the roadside. So you can only cope by the help of God and by prayers. Do you think you can get some sleep when your child leaves the house at 6PM and you can’t find them until 3AM? You can’t!” (Caregiver) “And the parent also doesn’t get it easy at all. You’ll be worried and just be overthinking. Thinking about what the family needs and about providing food for them. And all that becomes extremely distressing when you also have yourself to take care of.” (Caregiver) “You see, when you see what your loved one is going through, you tend to question your abilities as a parent. You ask yourself if their situation is as a result of you not taking good care of them. I console myself with the fact that no parent in the whole world is perfect at parenting.” (Caregiver) “I was really distraught when my daughter was experiencing those symptoms because I thought I was going to lose her. I really cried. I cried a lot.” (Caregiver) “I’ve not gotten anyone saying why, but some people do say why me? Like why am I the only one in the situation? Like they feel like they’re the only one in the situation but like they are not so.” (Clinician)

#### Actions Taken to Address Early Psychosis

To address their own or their loved one’s early psychosis, it was common for patients and caregivers to seek help through religious or traditional means. Participants described seeking counsel from church leaders, visiting traditional or faith healing prayer camps (locations where prophets or church leaders house and provide faith healing services to individuals with mental and/or physical ailments) within their communities, and initiating herbal remedies. These steps often served as the initial action taken, delaying initiation of treatment within the formal health care system. One caregiver described starting to take action toward early psychosis support, *“*…when the child’s condition first came about, his mother took him to some churches and prayer camps which didn’t help. I didn’t like those places because some of the churches can deceive you and ask you to bring some money, buy all sorts of things which will result in no improvement in their health.” Clinicians also described patients arriving at APH following engagement with spiritual healing approaches. One clinician said, “…but the first point of contact is either a religious leader or the herbalist. And then if that fails…if there is an acute or an overwhelming agitation that they cannot control, that’s the time that they show up to us.” People often consulted their immediate social networks for help. One caregiver said, “I was there one day with a friend of mine [who] told me he knew someone who struggled with similar issues and when the person was brought to this hospital [APH], that person got better and is even married now. I then decided to discuss it with my child’s mother… She also agreed and we brought the child to this hospital.”

#### Sources of Early Psychosis Support and Information 

Patients and their caregivers described information and resources that were helpful in their journey to address early psychosis. Psychoeducation about psychosis and psychotic disorders, treatment options, and success stories was helpful to those impacted by early psychosis. One patient said, “I went to research about the sickness, schizophrenia…so I had knowledge about the sickness…And it has helped.” A clinician described the usefulness of psychoeducation, “So with interacting with people, patients with early psychosis, one thing I noticed is that most of them are confused as to what they are experiencing. Because it’s something new to them. So you have to take time to try to explain to them the symptoms and what they are going through.”

#### Role of Caregivers in Early Psychosis Care

Caregivers of people with early psychosis play vital roles in encouraging their loved ones to engage in care from the formal health care system; providing comfort, listening to their loved ones’ experiences, and strategizing about next steps; providing material support via housing, transportation, and financial support; and supporting medication adherence. Often, the extent and frequency of caregiver responsibilities associated with early psychosis care come at a cost to caregivers’ mental health and can contribute to burnout. One caregiver described being spread thin before getting help: “So when he started experiencing symptoms…we did so many things. I did so many things, because of his condition…someone pointed out this place [APH], a mental hospital where we could get some help…truly, I have seen an improvement.” Caregivers often provide medication adherence support. One clinician recounted, “…if the family members get to understand this and would help them to be taking their medications, providing them with the support to come for reviews, I mean eventually…we will see a change in their conditions.”

#### Toll of Early Psychosis Caregiving on Caregivers

Providing comfort, listening, and strategizing about next steps for their loved ones’ treatment was a key caregiver role described by participants. One caregiver said, “What helped us decide to take action was love. And a parent wouldn’t just stand by while their child suffers.*”* Participants consistently described caregivers providing vital material resources such as housing, transportation to mental health appointments, and financial support. Demonstrating the financial support required, one caregiver said, “This is the phone I use. Only this one. I can’t go buy an expensive phone when my child has a mental disorder.”

Actions taken to address early psychosis often take a toll on caregivers in the form of mental stress, burnout, and financial insecurity. One clinician described, “…some people do say ‘why me?’ Like ‘why am I the only one in the situation?’” A caregiver described the hardships faced by parents of patients with early psychosis, *“*…the parent doesn’t get it easy at all. You’ll be worried and just be overthinking. Thinking about what the family needs and about providing food for them. And all that becomes extremely distressing when you also have yourself to take care of.” Another caregiver said, *“*…you tend to question your abilities as a parent. You ask yourself if their situation is as a results or you not taking good care of them.”

### Barriers and Facilitators to Psychiatric Early Psychosis Care in Ghana

Focus group discussions with patients with early psychosis, caregivers, and clinicians illuminated several barriers that prevented families from seeking or achieving formal health care system–based early psychosis care. These discussions also highlighted motivating factors that facilitated facility-based care.

#### Barriers

Stigma related to receiving care at a psychiatric hospital was a commonly reported barrier to seeking care at APH or other formal health care facilities. Caregivers and patients described having to overcome their own negative beliefs about receiving care at APH. A caregiver said, *“*…my son after finding out I was bringing him here got aggressive and didn’t want to come. He had to be boxed in in the taxi by three people...But after talking with the psychologist, he calmed down and admitted this wasn’t a place for ‘mad’ people.” Caregivers, while grateful for the care received at APH, expressed preference for the hospital to no longer be called a “psychiatric” hospital. One caregiver reported, “The name of this place, that’s ‘Psychiatric Hospital,’ like a hospital for mentally ill persons. I think polishing or changing the name would be a good thing. Because there’s a lot of stigma surrounding the psychiatric hospital.”

Lack of prior knowledge about psychosis symptoms, mental health conditions, or APH often served as a barrier to seeking facility-based early psychosis care. One clinician said, “They don’t believe this is what is going to work…they are skeptical because they don’t believe…it’s a medical problem.” Another clinician reported, “…most of them are committed to superstitious things or religious-connected, religious problems, but not a mental health problem.” Further, advice from within the community encouraging patients and caregivers to seek care from faith-based institutions also served as a barrier to facility-based care. This sometimes resulted in long delays before patients eventually sought care at APH. One caregiver recounted, “He experienced the symptoms for about two years before we brought him here. That was because people had convinced me to think it was a spiritual problem.”

Practical limitations such as financial burden associated with medications and facility-based services, logistical factors, distance from the health facility, and lack of trust in the health care system capacity additionally served as barriers to facility-based early psychosis care. Patients expressed concern about their caregivers’ ability to afford their treatment in the context of multiple financial obligations and limited finances. One patient reported, “If not for financial challenges I would’ve gotten help here way earlier than I did.” Caregivers also expressed difficulty in financing transport and treatment for their loved ones. Clinicians described health care system resource limitations and their impact on patient experiences. One clinician said, “Sometimes you see a patient and you look at the progress you are making, you want to see the person probably the next week or the following week but then you…realize that the date you want to give them is fully booked …That kind of affects the efficacy of the therapy that we do for them.”

#### Facilitators

Several factors motivated patients with early psychosis and their caregivers to seek facility-based early psychosis care. Prior knowledge or awareness about psychosis symptoms, mental health conditions, or APH sometimes encouraged facility-based care-seeking. One caregiver said, “We took him to church, and someone pointed out this place [APH]…saying he got the help he needed here, so he believed my son would also get help here…truly; I have seen an improvement.” Clinicians described awareness-raising activities and familial experience with mental health as other avenues facilitating facility-based care. One clinician said, “During mental health awareness month, we see people trooping in. We ask them, ‘How do you hear about our services?’ And they would be like, ‘I heard about you on the radio, I heard about it [APH] on TV.’”

Patients with early psychosis and caregivers who participated in focus group discussions expressed appreciation for the professional and competent care they received at APH, particularly emphasizing trust in their providers. One patient said, “…the doctors were ready to take care of me. Every time I call them, I get through to them, and they call me too to check up on me.” Another patient said, “Okay, what made it easy was like this doctor, Dr. [name]. He was always at your beck and call…He was ready to listen, so I also told him a lot of things.” As discussed in a prior section, caregivers were a leading facilitator for seeking and continuing facility-based services. Family members often heavily influence a loved one’s initial care-seeking, financing, and logistical support, treatment adherence, and ultimately reintegration into the community.

### Opportunities for Digital Health for Early Psychosis

We explored opportunities for enhancing early psychosis care by leveraging technology. Among patients with early psychosis, caregivers, and clinicians, we asked about their use of technology to support their typical daily activities, their use of technology to support psychoeducation or mental health, and their perceptions and feelings about the potential use of technology to support mental health. Common uses of technology in the daily lives of patients with early psychosis and caregivers included internet access for searching information, communicating with friends and family, and using social media. Extended and additional quotations about current care practices for early psychosis in Ghana are provided in Table 3.

**Table 3.** Illustrative quotations about opportunities for digital mental health for early psychosis*.*

Unhelpful	Helpful
Typical interactions with technology in daily life	
(Patient) Uh once you even get the phone, you need money to maintain it you need money to maintain it and buy also data bundles or data plan that will allow you to surf the internet. So if you don’t have it’s like you’re just holding a white elephant. It just (1:23:03, inaudible). You understand what I’m saying?	(Caregiver) .we use the phone for countless things. It’s what the youth use to study. One of my sons in JHS 2 uses the phone for every school work, so the phone truly helps.
(Clinician) In addition to that, I’ve come to realize that some of the patients who experience especially insomnia, when you are trying to establish or come up with a sleep routine, just trying to understand their sleep behaviors, you notice that, they are up till like 2AM, 3AM What are you doing at that particular time? They will tell you mindless scrolling.	(Patient) For me, if I get a phone, I can use it to make phone calls and find a job. Things like that.
(Clinician) Because growing up, I mean, when you’re always on your phone, your parents might scold why are you always on your phone? Come out, come and do this blah blah blah. So although genuinely you might be on the phone seeking help for whatever condition you are going through but you maybe misconstrued by your older caregivers. They might scold you for always being on your phone.	(Caregiver) I’m a seamstress so I use the phone to get dress inspirations online.I store client orders on the laptop. The TV is mounted in the shop so I watch some shows to release stress. I also what the news or movies or other TV shows.
(Patient) Yeah, sometimes the network is slow. Um it makes it difficult for you to access certain things on the internet.	(Patient) Through the phones, the phones and the you can you can acquire for scholarships for schools outside the country.
(Patient) Lack of financial support. People don’t have the money or the resources to buy a mobile phone or a television or a laptop. They won’t be updated on what is going on the internet.	(Caregiver) I use this same phone to update two of my friends about my son’s condition and ask them to help him find a job. So if I have a phone, (1:11:35 - 1:11:44, inaudible). I use the phone for business. I’m a carpenter so I get booked over the phone. The phone helps with communication.
Experiences using technology to support mental health	
(Clinician) You know also I think that, that is the beauty of human, humanness. When it’s totally technology it’s almost robotic but then when you as a human or like professionals are able to consume that information and artistically tweak it to make it more relatable to the client. That’s better than just consuming and then transferring robotic information.	(Caregiver) The little I’ll add on is uh honestly, the phone really helps.It helps because when necessary, I can contact the doctor from the comfort of my home. The doctor will call me back if he’s busy. The psychologist as well. So the phone is very necessary for communication And for things related to my child’s mental health condition.
(Clinician) Male participant: So, sometimes I think they have their. they have their opinions, and they want them validated. So they, Uh, sort of Uh just pick out what will push what they want to say.	(Clinician) So with regards to the phones, I think some patients are able to contact me on the phone. And um sometimes they have complaints that ordinarily they wouldn’t be able to come for reviews to you know talk about it. So we talk about it over the phone.
(Clinician) I think my reservation is the tendency it has to replace it [in-person mental health care] instead of complement it. So it’s supposed to be a tool to complement our services, to complement our work. But it runs the risk of patients replacing it instead. .It’s stands the risk of doing the opposite of what we intend it to do.	(Clinician) I think in the last couple of years, the advocacy around mental health has gone up a notch. So that helps as well. Because I remember back in medical school, a couple of colleagues and myself took it upon ourselves to do some kind of mental health awareness on social media, including YouTube.
(Clinician) One challenge that we particularly had to struggle with is internet connectivity. So the tool we are using is based on internet. In our part of the world, we don’t have very fast internet. So there are so many interruptions.And then, also, even when the internet is available, it comes at a high cost.	(Patient) Female participant 1: As for me, I used the TV. Sometimes I watch Nigerian movies with moral lessons that make you calm and happy. So that’s what I often use. Male translator: Do those movies include situations similar to your condition? Female participant 1: Yes. Sometimes, I see things that I’ve also experienced so when that happens, I pick a few things from the movie, which reduces overthinking.
Perceptions and feelings about digital mental health	
(Clinician) So usually they will attribute apps to being foreign.there are a lot of conspiracy theories about apps and you know downloading stuff on your phone.These are people who already have some delusions, some people, I mean those are some of the cultural things that are going to. play (out) when it comes to using apps.	(Patient) It can also help you get more information about your health or some remedies or care.
(Clinician) I was just worried of the legality, like legal issues when it comes to our digital care. Like, will the physician take that responsibility or duty of care? Because if the client should do something wrong from the app, what will be your responsibility (as a clinician)? Those are the things I’m also worried about.	(Caregiver) You guys can educate us on the drugs prescribed so that we can search and know exactly what the drug looks like before purchase.
(Clinician) If I have to do reading everything, going through the menu, having to scroll through it and then download the documents and have to do everything all together, it’s really very difficult for our clients to want to engage with such apps.	(Clinician) Personally, I think there are a lot of pitfalls in treatment and barriers we face .I think for me it’s interesting to see the merge; how technology can bridge the gap in our treatment plans and help with like seeing patients and um trying to break the barrier basically.
(Clinician) And sometimes in that regard [using a phone for providing mental health care], it’s hard to put certain boundaries. Maybe initially it helps. You’re able to follow up, make sure the patient comes back for reviews. Then the patients, when it’s time to go off that medium, they insist on still remaining there.	(Clinician) I just quite excited about the fact that it might make my clients’ life easier in terms of having to come to the hospital every time they are feeling some distress or having to call somebody they might not reach. I just feel like digital mental health interventions would make their journey to recovery much easier and it’ll also prevent a relapse like much better than if you keep coming and going.

#### Experiences Using Technology to Support Mental Health

Patients with early psychosis and caregivers found technology helpful in supporting their search for information about mental health conditions and communicating with care providers. Caregivers highlighted the convenience of using their phones to reach out to providers from their homes. This sentiment was supported by clinicians, who noted that technology helps caregivers share concerns or ask questions that otherwise might not be expressed. One clinician said, “…some patients are able to contact me on the phone... sometimes they have complaints that ordinarily they wouldn’t be able to come for reviews [to address].*”* Participants also reported using technology to manage mental distress. One early psychosis patient said, “You also get some kind of reassurance when you listen to songs or sermons on the radio.*”* Another patient said, “Sometimes I watch Nigerian movies with moral lessons that make you calm and happy…I see things that I’ve also experienced so when that happens, I pick a few things from the movie, which reduces overthinking.*”*

#### Potential of Digital Health to Address Barriers

Generally, participants perceived that digital health for early psychosis could improve understanding of symptoms, enhance mental health support between facility-based visits, and encourage continuity of care. One caregiver noted that a digital support tool could reduce challenges with identifying authentic medications: *“*…you guys can educate us on the drugs prescribed so that we can search and know exactly what the drug looks like before purchase.” One clinician felt that introducing a digital support tool could reduce cultural stigma around mental health: “…more advocacy for specific psychosis cases and creating awareness about what exactly goes into psychosis would be a very good start toward minimizing the stigma and then promoting people’s health-seeking behaviors.”

#### Concerns About Implementing Digital Mental Health

Early psychosis clinicians also expressed some negative perceptions about potential digital approaches. In contrast to the clinician who felt a digital tool could support continuity of care, another felt digital approaches could influence discontinuation of care. One clinician said, “Maybe initially it helps. You’re able to follow up, make sure the patient comes back for reviews. Then the patients, when it’s time to go off that medium [technology], they insist on still remaining there.” Another clinician was concerned that digital tools could worsen delusional beliefs, *“*…there are a lot of conspiracy theories about apps and you know downloading stuff on your phone…These are people who already have some delusions…those are some of the cultural things that are going to… play (out) when it comes to using apps.” Clinicians also emphasized the importance of a thoughtful introduction of a digital intervention for early psychosis by appropriate stakeholders to encourage trust and dispel perceptions of malintent: “It depends on who has accepted it and who is propagating it. If it’s even a good intervention, but it’s brought in by government that is leading it, it will typically be termed as ‘oh, it is this political party and they are using it for this agenda to get votes.’”

Overall, participants remained hopeful about additional early psychosis support through a digital intervention. A caregiver said, “We will embrace knowledge or education on the use of technological devices.”

## Discussion

### Principal Findings

In this qualitative study among patients with early psychosis, their caregivers, and their mental health care providers in Ghana, several themes emerged, exemplifying opportunities to enhance early psychosis care with digital mental health interventions (eg, smartphone-based apps, text-messaging interventions, telehealth interactions, and so on). Early psychosis care in the West African region is often delayed due to care-seeking from spiritual and traditional healing approaches and impeded or discontinued by practical barriers (eg, financial, logistical, real, or perceived health care system capacity limitations), as identified in other studies in Africa [37]. Substantial engagement by caregivers in early psychosis care is common in Ghana, which often vitally facilitates treatment yet imposes caregiver burnout and mental distress, as reported in other settings [38].

Youth experiencing their first episode of early psychosis in LMIC settings of West Africa represent a particularly vulnerable population. However, early intervention with effective treatment has the potential to improve their symptom trajectory, functioning dramatically, and ultimately integration in society [10,39]. Digital interventions to support early psychosis treatment are expanding globally, with demonstrated feasibility, acceptability, and preliminary efficacy to improve treatment outcomes [40], yet they do not currently exist in West Africa. Existing tools have strengths in leveraging evidence-based psychotherapies for early psychosis symptom management and treatment adherence promotion; however, they are not appropriate for West African contexts due to high literacy requirements and cultural irrelevance. Our study is one of the first to characterize patient, caregiver, and clinician perspectives about the use of digital interventions to address early psychosis in the African region. Focus on early intervention in psychosis programs in LMICs is generally low [41]. Our qualitative findings identify potential integration points, content, attributes, and unique strengths of digital modalities that could be leveraged to support patients with early psychosis and their caregivers in Ghana. Specifically, a tool that incorporates psychoeducation about early psychosis symptoms, effective treatment options, principles of evidence-based psychotherapies for self-management, and caregiver resources, integrated into in-person early psychosis care, could improve support for families in this context.

### Digital Health Opportunities Within Current Early Psychosis Care Practices

In qualitative discussions, patients with early psychosis, caregivers, and clinicians illuminated persistent gaps in current early psychosis care in Ghana that could be alleviated with digital mental health interventions. Delays in seeking treatment were common, often shaped by a misunderstanding of symptoms and a lack of awareness of available mental health services. Overwhelmingly, participants described a dearth of information and resources within the community about mental health. Longer duration of untreated psychosis in LMICs compared to higher-income countries is well-documented [14,42]. Digital mental health awareness campaigns and media about the signs of early psychosis and available mental health services could be offered via frequented platforms such as WhatsApp (WhatsApp Inc), YouTube (YouTube LLC), and social media sites to normalize experiences and encourage earlier treatment engagement.

Participants discussed that psychoeducation at the point of first contact was especially important in improving their understanding of mental health conditions and justifying the importance of timely and consistent treatment. A digital platform offering early psychosis psychoeducation could reinforce information shared during clinical visits and provide patients and families with an accessible resource between appointments. Digital psychoeducation integrated into early psychosis care has been shown to enhance quality of care and support patient engagement and functional recovery in high-income settings [43]. Both patients and caregivers described challenges navigating and sustaining engagement with psychiatric services. Digital tools could be used in these settings to support mental health referral or appointment tracking, care navigation, treatment adherence support, and shared decision-making among families impacted by early psychosis, which could strengthen continuity of care. Digital care navigation is increasingly implemented globally to facilitate mental health care system engagement and retention [44].

The extensive role that caregivers play in facilitating early psychosis treatment in this setting suggests that digital interventions for early psychosis will be most effective if they integrate caregiver-specific content alongside patient-focused modules. In-person mental health services have strengths such as the ability to distribute medications, conduct necessary labs and symptom screenings, enable real-time clinical observation and risk assessment, and provide structure and social connection. Digital interventions integrated into routine care could leverage the strengths of in-person care while improving consistency and completeness of psychoeducation and therapeutic messages about early psychosis. Cultural values of family cohesion and community shared responsibility are assets in West Africa [45-47], contributing to high potential for early psychosis remission and functional recovery if caregivers are provided ample psychoeducation and resources in this setting. Caregivers in Ghana described significant emotional and financial stress, similar to other settings [38,45], underscoring the potential benefit of providing caregiver-specific content (eg, coping strategies, resources, and self-care approaches) via digital interventions to support the well-being of caregivers and their loved ones.

Our findings were specifically evaluated in the context of early psychosis, yet they likely have applicability to other mental health disorders. The first episode or early stage of psychosis is a crucial time for intervention with high potential to improve symptom trajectory. Thus, our findings about the potential for digital tools to fill current gaps in early intervention in Ghana are particularly salient for early psychosis. However, we note that findings about intervention content (psychoeducation, care navigation and tracking, and caregiver resources) and barriers to mental health care that could be overcome by digital tools likely apply to other mental health conditions (eg, depression, anxiety, bipolar disorder, and so on) in West Africa.

### Digital Health Opportunities to Overcome Barriers and Leverage Facilitators of Facility-Based Early Psychosis Care

Participants described barriers to care-seeking and continuity within the mental health care system, including stigma around mental illness, spiritual or supernatural beliefs about symptoms, limited knowledge of psychosis and available care, financial and logistical challenges such as cost, distance, and long wait times, and lack of insight or reluctance to seek treatment. These findings are consistent with prior research in West Africa about barriers to mental health care [15,48,49]. Motivators for seeking or continuing care included positive experiences and trust in mental health care providers, success stories from community members, family support, and increased awareness of psychosis and treatment options.

Digital mental health interventions could supplement facility-based care, offering opportunities to overcome barriers while amplifying care facilitators. A commonly cited factor delaying facility-based early psychosis care was the attribution of psychosis symptoms to spiritual causes [15,48]. Digital interventions hold promise for counteracting misinformation about early psychosis in Ghana by integrating spiritual explanations for psychosis with messages that promote evidence-based etiologic understanding [21]. Further, participants expressed hesitancy to seek care at APH due to stigma historically associated with the institution as a former “lunatic asylum” under colonial rule [49]. Participants emphasized the importance of normalizing and destigmatizing mental health conditions in Ghana, which could be facilitated via digital content relevant to the cultural context. A recent meta-analysis demonstrated stigma-reduction effects of online interventions among people with mental health conditions [50]. Content delivered privately via digital modalities may reduce shame and foster acceptance. The on-demand and location-agnostic nature of digital interventions may circumvent common practical barriers to care, including transportation costs, long wait times, and extensive travel distances. Accessing psychoeducational materials in the privacy of one’s home may encourage repeated engagement and deeper reflection, supporting the development of insight among patients with early psychosis.

Leveraging existing facilitators within the formal health care system–based care, digital interventions are an optimal modality to extend patient-provider communication and amplify trust-building. Expansion of telehealth in psychiatry accelerated during the COVID-19 pandemic to address barriers to in-person care, exemplifying the potential for digital approaches to improve patient-provider alliance [51]. Digital interventions could also include success stories as motivating examples, amplifying one of the approaches that clinicians voiced as effective in engaging and retaining families in treatment. Additionally, providing caregiver-focused content in a digital intervention could harness the integral influence of caregivers in facilitating early psychosis care, potentially expanding that influence and providing caregiver-specific well-being support.

### Digital Health Opportunities Based on Participant Perceptions and Experiences

Focus groups with patients with early psychosis, caregivers, and clinicians concluded with discussions about their experiences with technology and perceptions of using technology to support early psychosis care. Our findings highlight the widespread use of phones for information seeking and communication—both to support typical daily tasks and specific mental health–related objectives. The ubiquity of mobile phone availability and the acceptability of digital mental health interventions for early psychosis were echoed in our team’s survey results among more than 250 individuals affected by early psychosis in Ghana [36]. A digital early psychosis intervention could be embedded within this familiar hardware to improve uptake and usability. Participants also described seeking relaxation and stress reduction via digital content types (eg, radio sermons and television shows with moral messages). Digital tools could provide evidence-based psychoeducation and psychological skills tutorials relevant to early psychosis using culturally acceptable narrative storytelling formats [52]. Clinicians emphasized the importance of introducing a digital intervention via trustworthy, respected, and nonpartisan community leaders to optimize trust and uptake. Participants further emphasized that a digital intervention should be accessible offline in settings with low internet bandwidth and should use simple interfaces and limited written language to optimize equity.

### Limitations

This qualitative study enrolled a small sample of patients with early psychosis, caregivers, and clinicians at APH. As such, participants were enrolled based on their care attendance at a single hospital in Accra, Ghana. Our findings may not be generalizable to patients not seeking facility-based care or those attending care in other facilities in the region. However, we intentionally situated our study at APH, the largest and most prominent psychiatric hospital in Ghana. Eligibility for patients with psychosis was based on receiving an initial psychosis diagnosis within the prior 5 years without continuous treatment exceeding 3 months. This definition aligned with that used by APH’s early psychosis pilot program and reflects the long duration of untreated psychosis commonly observed in Ghana. This definition may be overly inclusive, such that it includes individuals who would not be considered “early psychosis” in other settings; however, our priority was alignment with the care context at APH. Since qualitative research was conducted via group discussions, our study may have been subject to social desirability bias, whereby participants tailored their responses to align with perceived expectations or desired responses, particularly given the sensitive and stigmatized nature of the topic. To counteract this potential bias, trained qualitative researchers opened each focus group by reinforcing the voluntary nature of study participation and the researchers’ genuine interest in understanding participants’ authentic experiences and perceptions.

### Conclusions

Our qualitative study among those affected by early psychosis in Ghana highlights the potential for culturally grounded digital interventions to address acute gaps in early psychosis care in West Africa. By leveraging familiar technologies to disseminate psychoeducation, challenge stigmatizing beliefs, and support care continuity, digital tools may offer an acceptable and accessible complement to facility-based mental health services. Our findings suggest relevant content, design features, and contextual strengths that could be harnessed to develop a digital early psychosis intervention to support patients and their caregivers. Practical specifications such as offline-accessible, low-bandwidth, and low-literacy features are particularly relevant for digital mental health interventions in West African settings. As Ghana and other West African countries expand mental health service coverage, digital approaches represent a promising avenue to strengthen support for families affected by early psychosis. Overall, individuals affected by early psychosis in Ghana are open to digital interventions to enhance early psychosis treatment in West Africa. Future research should build on these findings to co-develop culturally relevant and evidence-based digital interventions for early psychosis in the region.

## Abbreviations

APH: Accra Psychiatric Hospital
LMIC: low- and middle-income country
REDCap: Research Electronic Data Capture

## References

[1] (2020). Mental health ATLAS 2020. World Health Organization.

[2] Sankoh O, Sevalie S, Weston M (2018). Mental health in Africa. Lancet Glob Health.

[3] Ofori-Atta A, Read UM, Lund C (2010). A situation analysis of mental health services and legislation in Ghana: challenges for transformation. Afr J Psych.

[4] Esan O, Abdumalik J, Eaton J, Kola L, Fadahunsi W, Gureje O (2014). Mental health care in Anglophone West Africa. Psychiatr Serv.

[5] Malla AK, Norman RMG, Joober R (2005). First-episode psychosis, early intervention, and outcome: what have we learned?. Can J Psychiatry.

[6] Fusar-Poli P, McGorry PD, Kane JM (2017). Improving outcomes of first-episode psychosis: an overview. World Psychiatry.

[7] Kane JM, Robinson DG, Schooler NR (2016). Comprehensive versus usual community care for first-episode psychosis: 2-year outcomes from the NIMH RAISE Early Treatment Program. AJP.

[8] Dixon LB, Goldman HH, Srihari VH, Kane JM (2018). Transforming the treatment of schizophrenia in the United States: the RAISE initiative. Annu Rev Clin Psychol.

[9] Correll CU, Galling B, Pawar A (2018). Comparison of early intervention services vs treatment as usual for early-phase psychosis: a systematic review, meta-analysis, and meta-regression. JAMA Psychiatry.

[10] Padma TV (2014). Developing countries: the outcomes paradox. Nature New Biol.

[11] Hunt X, Abdurahman H, Omobowale O (2022). Interventions for adolescents and adults with psychosis in Africa: a systematic review and narrative synthesis. Glob Ment Health.

[12] Fekadu A, Medhin G, Lund C (2019). The psychosis treatment gap and its consequences in rural Ethiopia. BMC Psychiatry.

[13] Abiodun OA (1995). Pathways to mental health care in Nigeria. Psychiatr Serv.

[14] Lilford P, Wickramaseckara Rajapakshe OB, Singh SP (2020). A systematic review of care pathways for psychosis in low-and middle-income countries. Asian J Psychiatr.

[15] Patel V (2011). Traditional healers for mental health care in Africa. Glob Health Action.

[16] Nicholas A, Joshua O, Elizabeth O (2022). Accessing mental health services in Africa: current state, efforts, challenges and recommendation. Ann Med Surg (Lond).

[17] Doyle R, Turner N, Fanning F (2014). First-episode psychosis and disengagement from treatment: a systematic review. PS (Wash DC).

[18] Morillo H, Lowry S, Henderson C (2022). Exploring the effectiveness of family-based interventions for psychosis in low- and middle-income countries: a systematic review. Soc Psychiatry Psychiatr Epidemiol.

[19] Andualem F, Melkam M, Tadesse G (2024). Burden of care among caregivers of people with mental illness in Africa: a systematic review and meta-analysis. BMC Psychiatry.

[20] Cham CQ, Ibrahim N, Siau CS (2022). Caregiver burden among caregivers of patients with mental illness: a systematic review and meta-analysis. Healthcare (Basel).

[21] Ben-Zeev D (2018). Mobile health for mental health in West Africa: the case for Ghana. Psychiatr Serv.

[22] Chibanda D (2017). The future of psychiatry in Africa-thinking outside the box. Lancet Psychiatry.

[23] Africa: five countries have near 100% mobile phone ownership (survey). Ecofin Agency.

[24] Pobee F, Jibril AB, Shaikh AA, Turginbayeva A, Issayeva Z, Mutanov G (2026). Driving digital transformation in government services in Ghana: a value-creation perspective. Measurement: Digitalization.

[25] Schlosser DA, Campellone TR, Truong B (2018). Efficacy of PRIME, a mobile app intervention designed to improve motivation in young people with schizophrenia. Schizophr Bull.

[26] Terp M, Jørgensen R, Laursen BS, Mainz J, Bjørnes CD (2018). A smartphone app to foster power in the everyday management of living with schizophrenia: qualitative analysis of young adults’ perspectives. JMIR Ment Health.

[27] Peck CE, Lim MH, Purkiss M, Foley F, Hopkins L, Thomas N (2020). Development of a lived experience-based digital resource for a digitally-assisted peer support program for young people experiencing psychosis. Front Psychiatry.

[28] Bonet L, Torous J, Arce D, Blanquer I, Sanjuán J (2021). ReMindCare, an app for daily clinical practice in patients with first episode psychosis: a pragmatic real‐world study protocol. Early Intervention Psych.

[29] McCool J, Dobson R, Whittaker R, Paton C (2022). Mobile health (mHealth) in low- and middle-income countries. Annu Rev Public Health.

[30] (2021). Global Strategy on Digital Health 2020-2025.

[31] Sekhon M, Cartwright M, Francis JJ (2017). Acceptability of healthcare interventions: an overview of reviews and development of a theoretical framework. BMC Health Serv Res.

[32] Clarke V, Braun V (2014). Encyclopedia of Critical Psychology.

[33] Fereday J, Muir-Cochrane E (2006). Demonstrating rigor using thematic analysis: a hybrid approach of inductive and deductive coding and theme development. Int J Qual Methods.

[34] Shenton AK (2004). Strategies for ensuring trustworthiness in qualitative research projects. EFI.

[35] Creswell JW, Plano Clark VL (2017). Designing and Conducting Mixed Methods Research.

[36] Larsen A, Agorinya J, Beaulieu A (2025). Digital health for early psychosis in Ghana: patient and caregiver needs and preferences. Schizophrenia (Heidelb).

[37] Komu CK, Ngigi M, Melson AJ (2025). Barriers and facilitators to accessing mental health services for adults in sub‐Saharan Africa: a systematic review. Mental Health Science.

[38] Onwumere J, Lotey G, Schulz J (2017). Burnout in early course psychosis caregivers: the role of illness beliefs and coping styles. Early Interv Psychiatry.

[39] Reading B, Birchwood M (2005). Early intervention in psychosis: rationale and evidence for effectiveness. Dis Manag Health.

[40] Rus-Calafell M, Schneider S (2020). *Are we there yet?!*-a literature review of recent digital technology advances for the treatment of early psychosis. Mhealth.

[41] van der Ven E, Yang X, Mascayano F (2025). Early intervention in psychosis programs in Africa, Asia and Latin America; challenges and recommendations. Camb prisms Glob ment health.

[42] Mossaheb N, Schloegelhofer M, Kaufmann RM (2013). Duration of untreated psychosis in a high-income versus a low- and middle-income region. Aust N Z J Psychiatry.

[43] Vignapiano A, Monaco F, Panarello E (2025). Digital interventions for the rehabilitation of first-episode psychosis: an integrated perspective. Brain Sci.

[44] Jaso-Yim B, Eyllon M, Sah P (2024). Evaluation of the impact of a digital care navigator on increasing patient registration with digital mental health interventions in routine care. Internet Interv.

[45] Mbedzi TE, Van der Wath AE, Moagi MM (2024). The lifeworld of families of mental health care users in rural South Africa: a phenomenological study. S Afr J Psych.

[46] Gupta M, Bowie CR (2018). Family cohesion and flexibility in early episode psychosis. Early Intervention Psych.

[47] Koutra K, Vgontzas AN, Lionis C, Triliva S (2014). Family functioning in first-episode psychosis: a systematic review of the literature. Soc Psychiatry Psychiatr Epidemiol.

[48] Anjorin O, Wada YH (2022). Impact of traditional healers in the provision of mental health services in Nigeria. Annals of Medicine & Surgery.

[49] Ewusi-Mensah I (2001). Post colonial psychiatric care in Ghana. Psychiatr bull.

[50] Goh YS, Ow Yong QYJ, Tam WSW (2021). Effects of online stigma‐reduction programme for people experiencing mental health conditions: A systematic review and meta‐analysis. Int J Mental Health Nurs.

[51] Schubert NJ, Backman PJ, Bhatla R, Corace KM (2019). Telepsychiatry and patient-provider concordance. Can J Rural Med.

[52] De Vecchi N, Kenny A, Dickson‐Swift V, Kidd S (2016). How digital storytelling is used in mental health: a scoping review. Int J Mental Health Nurs.

